# *Lagerstroemia Speciosa* (L.) Pers Leaf Extract Attenuates Lung Tumorigenesis via Alleviating Oxidative Stress, Inflammation and Apoptosis

**DOI:** 10.3390/biom9120871

**Published:** 2019-12-12

**Authors:** Amria M. Mousa, Nermin M. El-Sammad, Abeer H. Abdel-Halim, Nayera Anwar, Wagdy K. B. Khalil, Mahmoud Nawwar, Amani N. Hashim, Elsayed A. Elsayed, Sherien K. Hassan

**Affiliations:** 1 Biochemistry Department, National Research Centre, Dokki, Cairo 12622, Egypt; amria0101@gmail.com (A.M.M.); nerminelsamad@gmail.com (N.M.E.-S.); abeer.hamid@yahoo.com (A.H.A.-H.); sherien.kamal.hassan@gmail.com (S.K.H.); 2 Pathology Department, National Cancer Institute, Cairo University, Cairo 12796, Egypt; 3 Cell Biology Department, National Research Centre, Dokki, Cairo 12622, Egypt; 4 Phytochemistry and Plant Systematics Department, National Research Centre, Cairo 12622, Egypt; 5 Zoology Department, Bioproducts Research Chair, College of Science, King Saud University, Riyadh 11451, Saudi Arabia; 6 Chemistry of Natural and Microbial Products Department, National Research Centre, Cairo 12622, Egypt

**Keywords:** benzo(a)pyrene, lung cancer, *Lagerstroemia speciosa*, oxidative stress, inflammation, apoptosis

## Abstract

One of the major etiological factors that account for lung cancer is tobacco use. Benzo(a)pyrene [B(a)P], one of the main constituents of tobacco smoke, has a key role in lung carcinogenesis. The present study was conducted to investigate the cytotoxicity of an aqueous ethanolic extract of *Lagerstroemia speciosa* (L.) Pers leaves (LLE) on human lung adenocarcinoma cells (A549), as well as its in vivo antitumor effect on a lung tumorigenesis mice model. Our results revealed that LLE possesses cytotoxic activity against the A549 cell line. Mice orally administered B(a)P (50 mg/kg body weight) showed an increase in relative lung weight with subsequent decrease in final body weight. Serum levels of tumor marker enzymes AHH, ADA and LDH and the inflammatory mediator NF-κB increased, while total antioxidant capacity (TAC) decreased. In addition, we observed the increased activity of metalloproteinases (MMP-2 and MMP-12) and levels of the tumor angiogenesis marker VEFG and the lipid peroxidation marker MDA, as well as decreased levels of the non-enzymatic antioxidant GSH and enzymatic antioxidants CAT and GSH-Px in lung tissues. Moreover, B(a)P administration up-regulated the expression of the COX-2 gene, pro-inflammatory cytokines TNF-α and IL-6, and an anti-apoptotic gene Bcl-2, and at the same time down-regulated expression of pro-apoptotic genes BAX and caspase-3 and the p53 gene. Pre- and post-treatment with LLE (250 mg/kg body weight) attenuated all these abnormalities. Histopathological observations verified the protective effect of LLE. Overall, the present data positively confirm the potent antitumor effect of *L. speciosa* leaves against lung tumorigenesis.

## 1. Introduction

Cancer is a major public health problem worldwide, and ranks as the second leading cause of death in many countries [1]. Lung cancer is the most prevalent and fatal cancer worldwide. According to the World Health Organization (WHO) [2], lung cancer accounts for about 2.1 million new cases and 1.8 million deaths per year. The most serious risk factor for lung cancer is cigarette smoke, which contains more than 69 established chemical carcinogens [3]. Benzo(a)pyrene, a polycyclic aromatic hydrocarbon, is the major carcinogen found in cigarette smoke and plays an important role in lung carcinogenesis through the induction of free radicals, stimulation of cell proliferation, and promotion of DNA adduct formation [4]. Khan et al. [5] and He et al. [6] reported that poor prognosis of lung cancer and appearance of symptoms at a relatively late stage, together with early occurrence of distant metastases, are the main reasons for lack of progress in treatments, putting lung cancer beyond the stage of conventional therapeutic options. The use of novel synthetic or natural compounds to prevent or reverse lung carcinogenesis is an emerging approach that could provide great benefit to public health.

Medicinal plants are a promising source of alternatives to pharmaceutical drugs in cancer therapy [7]. In recent years, phytochemicals have attracted immense interest as effective chemopreventive agents against cancer due to their powerful antioxidant activity [8,9], and a considerable percentage of anticancer drugs used today are of plant origin [10]. *Lagerstroemia speciosa* (L.) Pers (Lythraceae), a deciduous tropical tree commonly known as banaba, possesses several polyphenolic compounds [11]. To date, more than 40 phytoconstituents have been isolated and identified from the *L. speciosa* leaves. These phytoconstituents include ellagic acid and its derivatives, triterpenes, tannins, a triterpenoid, corosolic acid, quercetin, isoquercitin, flavones and glycosides, with various biological activities [12]. Traditionally, tea made from banaba leaves has been used to treat diabetes mellitus in Southeast Asia [13]. Recently, it has been reported that *L. speciosa* leaf extract has extensive antidiabetic, antiobesity [14], anti-inflammatory, antioxidant, antiviral, antibacterial [12], anti-hypertensive [15], antifibrotic [16] and analgesic [17] effects.

Isolated phytoconstituents from *L. speciose*, coupled with its biological activities and traditional uses, make *L. speciosa* a therapeutic candidate for further investigation. To date, there is no scientific evidence in the literature confirming the anti-cancer efficacy of *L. speciosa* leaf extract. In view of the abovementioned facts, aqueous ethanolic extract of *L. speciosa* leaves was chosen for this research to evaluate the in vivo antitumor effect on B(a)P-induced lung tumorigenesis in Swiss albino mice, as well as the in vitro cytotoxic activity towards a human lung adenocarcinoma cell line, A549.

## 2. Materials and Methods 

### 2.1. Plant Material and Preparation of Plant Extract

Fresh samples of *L. speciosa* leaves were collected from Zoharea Garden, Egypt. Plant authentication was carried out by Prof. Salwa Quashti, NRC, Cairo, Egypt. A voucher specimen (M 146) has been deposited at the herbarium of the NRC. *L. speciosa* leaves dried in shadow were crushed and exhaustively extracted with 70% (*v*/*v*) aqueous ethanol (EtOH) under reflux. The obtained eluent was dried under vacuum at 55–60 °C, then dissolved in EtOH to give an aqueous ethanolic extract of *L. speciosa* that was used in the present study.

### 2.2. Phytochemical Screening

The phytochemical screening of *L. speciosa* leaf extract (LLE) was performed for terpenoids, steroids, alkaloids, flavonoids, saponins, tannins, phenolic acids, glycosides, carbohydrates and anthraquinones, as described by Sofowora [18].

### 2.3. Determination of Total Phenolic Content

The total phenolic content of LLE was estimated by the Folin–Ciocalteu method as described by Ainsworth and Gillepsie [19]. A volume of 500 µL of LLE (1 mg/mL) was mixed with 2.5 ml of Folin-Ciocalteu reagent (diluted 10-fold with purified water) and 2 mL of 7.5% sodium bicarbonate (NaHCO_3_). The mixture was incubated at 45 °C for 15 min. Absorbance was measured at 765 nm against blank with a UV-Vis spectrophotometer. Gallic acid was used as a standard, and results were expressed as milligrams of gallic acid equivalent (GAE) per gram of dry extract. Values are presented as means of triplicate analyses.

### 2.4. Determination of Total Flavonoid Content

The total flavonoid content was estimated by the aluminum chloride (AlCl_3_) colorimetric method according to Brighente et al. [20]. A volume of 0.5 mL 2% AlCl_3_ in methanol was mixed with an equal volume of LLE solution (1 mg/mL). After 1 h incubation at room temperature, absorbance was measured at 415 nm against blank with a UV-Vis spectrophotometer. Rutin was used as the standard, and results were expressed as milligrams of rutin equivalents (RUE) per gram of dry extract. Values are presented as means of triplicate analyses.

### 2.5. DPPH Radical Scavenging Assay

Free 2,2-diphenyl-1-picrylhydrazyl (DPPH) radical assay was carried out according to the method of Alnahdi et al. [21]. An aliquot of 50 µL of LLE was added to 5 mL of 0.004% ethanol solution of DPPH. The mixture was incubated at room temperature for 30 min. The absorbance was measured at 517 nm against blank using a UV-Vis spectrophotometer. Ascorbic acid was used as a reference standard. The scavenging activity of the DPPH radical was expressed as inhibition percentage I (%), which was calculated as follows:

I(%) = ( AC− AS/ AC ) × 100

in which AS is the absorbance of the tested sample, and AC is the absorbance of the control reaction (contains all reagents except the tested sample).

### 2.6. In Vitro Cytotoxic Assays

Working LLE solutions (0.0–1.0 mg/mL) were prepared in dimethyl sulfoxide (DMSO, (CH_3_)_2_SO) and were sterile-filtered using 0.22 µm sterile syringe filters (Millipore, Burlington, MA USA). The work was performed with human lung adenocarcinoma cells, A549, and normal non-tumorigenic human lung epithelial cells, BEAS-2B (Sigma-Aldrich Chemical Company, St. Louis, MO, USA). Cells were propagated in Dulbecco’s modified Eagle’s medium (DMEM) supplemented with 10% fetal bovine serum (FBS), 1% antibiotic/antimycotic solution and 3.6 g/L NaHCO_3_. Cell cultivation and preparation was performed as per our previously developed protocol [22,23].

#### 2.6.1. MTT Assay

The in vitro cytotoxic effects of LLE extract were evaluated using the standard MTT assay [24,25]. The method depends on the reduction of 3-(4,5-dimethylthiazol-2-yl)-2,5-diphenyl tetrazolium bromide (MTT) by mitochondrial dehydrogenases in living cells. After being trypsinized and washed, 100 µL of cell suspension were seeded into 96-well plates to give a concentration of 10^4^ cells/well. Plates were then incubated at standard conditions for 24 h. After cell adherence, medium was exchanged with fresh medium containing serial dilutions of LLE extract, and then incubated for another 24 h. Afterwards, MTT was added (10 µL/well, 5 mg/mL in PBS), and plates were placed into the incubator for 4 h. Supernatants were aspirated, and DMSO (200 µL) was added. The absorbance of the developed formazan was read at 550 nm using a microplate reader (Thermo Scientific, Waltham, MA, USA) and was correlated with the percentages of viable cells. IC_50_ values were deduced from the linear regression of the calibration curve.

#### 2.6.2. Neutral Red Uptake (NRU) Assay

The Neutral Red Uptake (NRU) assay depends on detecting lysosomal efficiency of absorbing neutral red stain (3-amino-7-dimethylamino-2-methyl-phenazine hydrochloride) in living cells, according to Borenfreund and Puerner [26]. The assay is performed as per our earlier developed protocol [27,28] and similarly to the MTT assay. However, after cells were exposed to LLE dilutions for 24 h, the medium was removed, and cells were washed twice with PBS and incubated for 3 h in media containing NR (50 µg/mL). Media was washed off rapidly with a 0.5:1.0% solution of CH_2_O:CaCl_2_. Then, plates were incubated for 20 min in a mixture of CH_3_COOH:C_2_H_5_OH solution (1:50%) to extract the dye. Plates were read at 540 nm.

In both MTT and Neutral Red Uptake (NRU) assays, results were compared with control sets, and run under identical conditions without the test compound. Furthermore, treated cells were microscopically examined for possible morphological changes using an inverted contrast microscope (Nikon Eclipse T500, Nikon, Tokyo, Japan) at 10 × magnification.

### 2.7. In Vivo Anticancer Activity

#### 2.7.1. Animals

Male Swiss albino mice weighing 22–25 g were obtained from the animal facility at the National Research Centre, Egypt. Animals were housed in polypropylene cages under room temperature and were acclimatized to 12 h light and dark cycles. Animals were fed with commercially-available standard pellets and were given water ad libitum. All animal experiments were designed and conducted according to the ethical norms of animal care in accordance with the Helsinki Declaration of 1975, as revised in 2000 and 2008 and approved by the Medical Research Ethics Committee of the National Research Centre (Cairo, Egypt) with ethical registration number 16/164.

#### 2.7.2. Experimental Design

**Figure 1:**
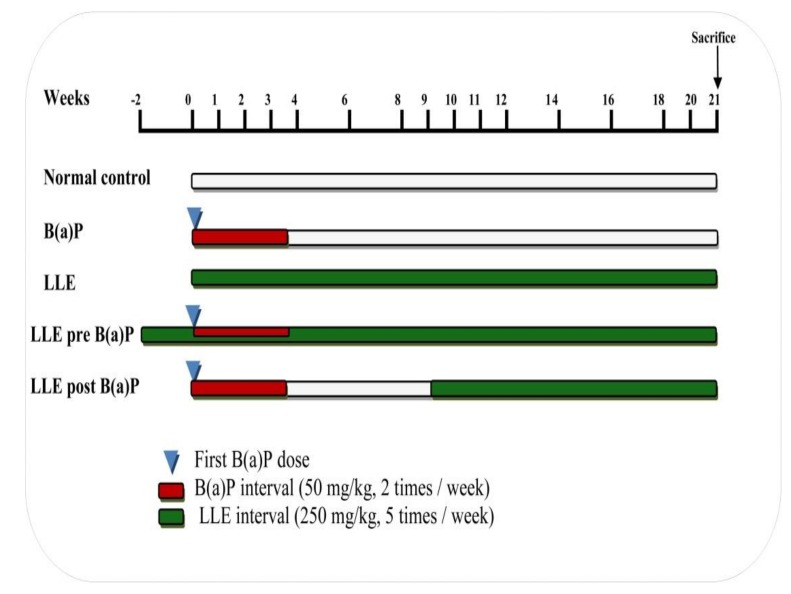
Experimental design of the study.

The dose of LLE (250 mg/kg body weight) was administered orally in accordance with Saumya and Basha [29], while the dose of B(a)P (50 mg/kg body weight, dissolved in corn oil) was administered orally in accordance with Kasala et al. [30]. Experimental animals were divided into five groups, each group composed of six animals. Treatment groups are shown in Figure 1 as follows:

- Normal control group: Mice were given corn oil through the experimental period.
- B(a)P group: Mice were administered B(a)P twice a week for four successive weeks to induce lung cancer by week 21.
- LLE group: Mice were administered *L. speciosa* leaf extract five days a week until the end of the experimental period.
- LLE pre-B(a)P group: Mice were pre-treated with *L. speciosa* leaf extract two weeks prior to the first B(a)P dose, and doses of both continued on the schedule described above until the end of the experimental period.
- LLE post-B(a)P Group: Mice were treated with *L. speciosa* leaf extract after week 9 following the first B(a)P dose, and doses of both continued on the schedule described above until the end of the experimental period.

Overnight-fasted animals were anesthetized with ether at the end of the experimental period. Blood was drawn from the retro-orbital sinus, and serum was obtained. Lungs were immediately removed from the animals and were washed in ice-cold saline. Tissues were weighed and sliced into three portions. Using a Tissue Master TM125 (Omni International, Kennesaw, GA, USA), one portion was homogenized in 0.1 M potassium phosphate (KH_2_PO_4_) buffer (pH 7.4), then centrifuged at 3000 rpm for 10 min at 4 °C. Supernatants were separated and used to analyze various biochemical parameters. The second portion was fixed in 10% formalin for histopathological studies. The last portion was quickly frozen at −80 °C to be used for molecular analysis. 

All images were acquired using a compound Nikon Eclipse E600 Binocular Microscope equipped with a Japanese Nikon Digital Camera (model DXM1200F) and fluorescent stereo microscope (Olympus ZX12) with DP72 camera.

### 2.8. Biochemical Analysis

#### 2.8.1. Antioxidant and Oxidative Stress Markers

Serum total antioxidant capacity (TAC) and glutathione peroxidase (GSH-Px) in lung tissues were estimated using kits from Biodiagnostic Research Reagents (Egypt). Reduced glutathione (GSH) in lung tissue was assayed at 412 nm according to the method of Beutler et al. [31] based on the yellow-colored complex formed by the reaction of GSH and 5, 5’-dithibis-2-nitrobenzoic acid (DTNB). Catalase activity was estimated at 240 nm by monitoring the disappearance of hydrogen peroxide (H_2_O_2_) according to the method of Aebi [32]. Malondialdehyde (MDA) was measured in lung tissues using the thiobarbituric acid-reactive substance (TBARS) assay according to the method of Lef’evre et al. [33]. The colorimetric reaction of the lipid peroxidation marker MDA with thiobarbituric acid (TBA) is quantified by the formation of pink chromogen complex and the absorbance was read at 535 nm.

#### 2.8.2. Serum Marker Enzymes

Aryl hydrocarbon hydroxylase (AHH) and adenosine deaminase (ADA) were assayed by an enzyme-linked immunosorbent assay (ELISA) using Glory Science Co., Ltd. kits (Glory Sci. Co. Ltd., Wujiang Jiangsu, China) in accordance with manufacturer instructions. Lactate dehydrogenase (LDH) was also assayed colorimetrically using kits from Spectrum Diagnostics (Egypt).

#### 2.8.3. Gelatinolytic Metalloproteinase (MMP-2) Activity

MMP-2 enzymatic activity was detected in lung tissue by gelatin zymography according to Frederiks and Mook [34]. This technique was carried out by electrophoresing homogenized lung tissue samples in sodium dodecyl sulfate-polyacrylamide gels (SDS-PAGE, 7.5%) copolymerized with gelatin (0.2%). 

After electrophoresis, the catalytic activity of MMP-2, reversibly inhibited by SDS, was restored by using 2.5% aqueous Triton X-100 then incubating gel in detection buffer (50 mM Tris-HCl, pH 7.5, and 5 mM CaCl_2_, 1 μM ZnCl_2_) for 20 h at 37 °C. The partially-renatured enzyme degraded the gelatin, leaving clear zones that were visualized by staining the gel with 0.5% Coomassie Brilliant blue R-250. Gelatinase activity relative to control samples was determined by densitometric scanning of the bands (Bio-Rad Gel Doc^TM^ XR+ System, USA) using Image Lab^TM^ 4.0 Software.

#### 2.8.4. Elastolytic Metalloproteinase Activity (MMP-12)

MMP-12 activity was performed in lung tissues with the use of *N*-succinyl-trialanyl-*p*-nitroanilide as the substrate, as described by Zay et al. [35].

#### 2.8.5. Tumor Angiogenic Marker

The vascular endothelial growth factor (VEGF), the most potent and predominant stimulator of tumor angiogenesis, was measured in lung tissue using Glory Science Co., Ltd. ELISA kits (Glory Sci. Co. Ltd., Wujiang Jiangsu, China).

#### 2.8.6. Serum Inflammatory Mediator

Nuclear factor kappa (NF-κB) was estimated using Glory Science Co., Ltd. ELISA kits (Glory Sci. Co. Ltd., Wujiang Jiangsu, China).

#### 2.8.7. Gene Expression Analysis

##### Total RNA Isolation

Total RNA was extracted from lung tissues using TRIzol® Reagent, (Invitrogen, Germany). First, one unit of RQ1 RNAse-free DNAse (Invitrogen, Darmstadt, Germany) was added to the isolated RNA to digest DNA residues. Total RNA was then re-suspended in diethyl pyrocarbonate-treated water and quantified spectrophotometrically at 260 nm. Total RNA purity was determined by measuring the absorbance at 260 and 280 nm, and the A260/A280 ratio was between 1.8 and 2.1. Aliquots were used for reverse transcription.

##### Reverse Transcription (RT) Reaction

Isolated poly(A)^+^ RNA was reverse-transcribed into cDNA using the RevertAid^TM^ First Strand cDNA Synthesis Kit (Fermentas, Germany). 5 µg total RNA was added to the master mix, which consisted of 50 mM MgCl_2_, 10 × RT buffer, 10 mM of dNTP, 50 µM oligo-dT primer, 20 IU ribonuclease inhibitor and 50 IU MuLV reverse transcriptase. The mixture was centrifuged at 1000× *g* for 30 s and transferred to the thermocycler (Applied Biosystems, Foster City, CA, USA). The RT reaction was carried out for 10 min at 25 °C, followed by 1 h at 42 °C, and terminated by a denaturation step at 99 °C, which lasted for 5 min. Afterwards, RT preparations were flash-cooled and used for cDNA amplification in the quantitative real-time polymerase chain reaction (qRT-PCR).

##### Quantitative Real Time-PCR (qRT-PCR)

A Step One Real-Time PCR machine (Applied Biosystems, USA) was used to determine the cDNA copy number. The PCR reaction was established in 25 μL reaction mixtures containing 12.5 μL SYBR**^®^** Premix Ex TaqTM (TaKaRa Biotech, Kusatsu, Shiga 525-0058, Japan), 6.5 μL distilled water, 5 μL cDNA template and equal volumes of 0.2 μM sense primer and antisense primer. The reaction program included the following stages: Stage one: 3 min at 95 °C. Stage two: 40 cycles, each containing 3 steps: 95 °C for 15 s, 55 °C for 30 s and 72 °C for 30 s. Stage three, carried out to obtain the melting curve: 71 cycles initiated at 60 °C, then increased by 0.5 °C every 10 s until 95 °C was reached. The oligonucleotide primer sequences of inflammatory and apoptotic genes are listed in Table 1. The quantitative values of the target genes were normalized on the basis of β-actin gene expression. Relative quantification of the target to the reference was calculated by the 2^−ΔΔCT^ method.

#### DNA Fragmentation Analysis

Apoptotic DNA fragmentation was analyzed by detecting the laddering pattern of nuclear DNA according to Lu et al. [40]. Briefly, lung tissues were homogenized, washed in PBS and lysed in 500 μl of DNA extraction buffer (50 mM Tris–HCl, 0.5% Triton X-100, 10 mM ethylenediaminetetraacetic acid (EDTA), and 100 μg/ml proteinase K, pH 8.0). After overnight incubation at 37 °C, the lysate was incubated with 100 μg/ml DNase-free RNase for 2 h at 37 °C, followed by three extractions with an equal volume of phenol/chloroform (1:1 *v*/*v*) then centrifugation at 15,000 rpm for 5 min at 4 °C. DNA was collected by precipitating the aqueous supernatant in two volumes of ice-cold absolute ethanol with 1/10 volume of 3 M sodium acetate, pH 5.2 at −20 °C for 1 h. The DNA pellet was then washed with 70% ethanol, air-dried and dissolved in 10 mM Tris–HCl/1 mM EDTA, pH 8.0. DNA was then electrophoresed on 1.5% agarose gel containing 0.5 μg/ml ethidium bromide. DNA fragments were visualized and photographed by exposing the gels to ultraviolet transillumination. A 100-bp DNA ladder (Invitrogen, Carlsbad, CA, USA) was included as a molecular size marker.

### 2.9. Histopathological Analysis

The formalin-fixed tissue samples were paraffin-embedded, thin-sectioned (5 μm), and mounted on microscope slides using standard histopathological techniques. Tissue sections were stained with hematoxylin and eosin (H&E) and examined under light microscopy (Olympus, Hachioji-shi, Tokyo, Japan).

### 2.10. Statistical Analysis

Statistical analysis was performed using the Statistical Package for the Social Sciences (IBM SPSS Statistics, version 19). Analysis of variance was performed by one-way ANOVA procedures. Significant differences between means were determined by the least significant digit (LSD) multiple range test at a level of *P* < 0.05. Data are expressed as mean ± standard error (SE).

## 3. Results

### 3.1. Phytochemical Screening

The present results (Table 2) show that the total phenolic content of LLE was (36.30 ± 1.07 mg GAE/g plant extract), and flavonoid content was (3.44 ± 0.59 mg RUE/g plant extract).

### 3.2. Radical Scavenging Activity of L. Speciosa

**Figure 2:**
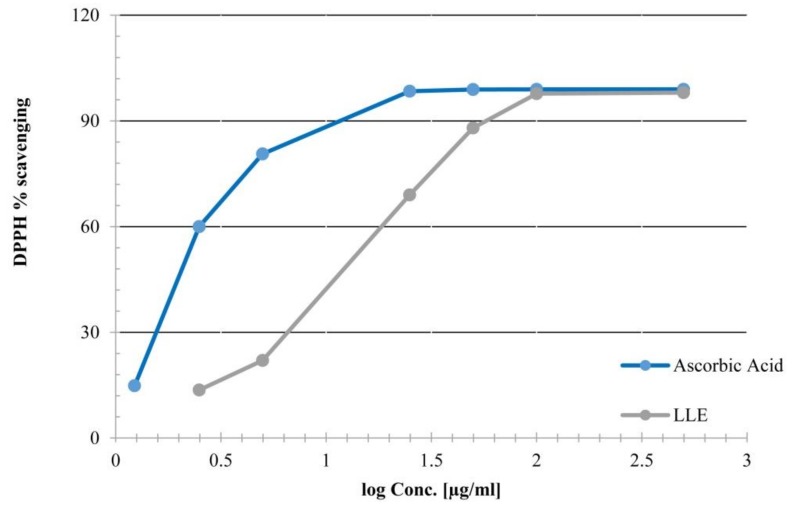
DPPH radical-scavenging activity of the aqueous ethanolic leaf extract of *L. speciosa*.

The DPPH radical scavenging assay was used to determine the antioxidant activity of LLE. Moreover, results presented (Figure 2) show that the percentage scavenging of DPPH radical for LLE was concentration dependent from 0.5 to 500 µg/mL (expressed in *log* scale). LLE gave 97.71% radical scavenging at a concentration of 100 µg/mL with ED_50_ value 10.21 ± 1.33 µg/mL compared to 1.83 ±1.41 μg/mL of ascorbic acid used as a standard antioxidant.

### 3.3. In Vitro Cytotoxic Activities

**Figure 3:**
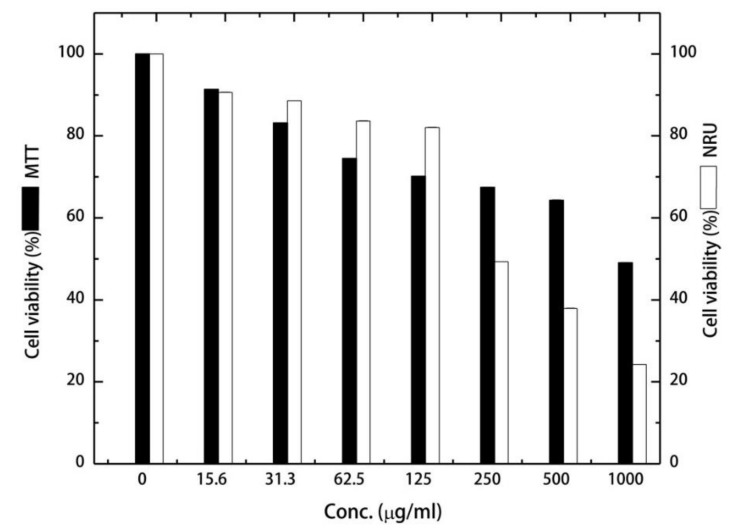
Effect of different concentrations of LLE on the viability of A549 lung adenocarcinoma measured by 3-(4,5-dimethylthiazol-2-yl)-2,5-diphenyltetrazolium bromide and Neutral Red Uptake (NRU) assays.

**Figure 4:**
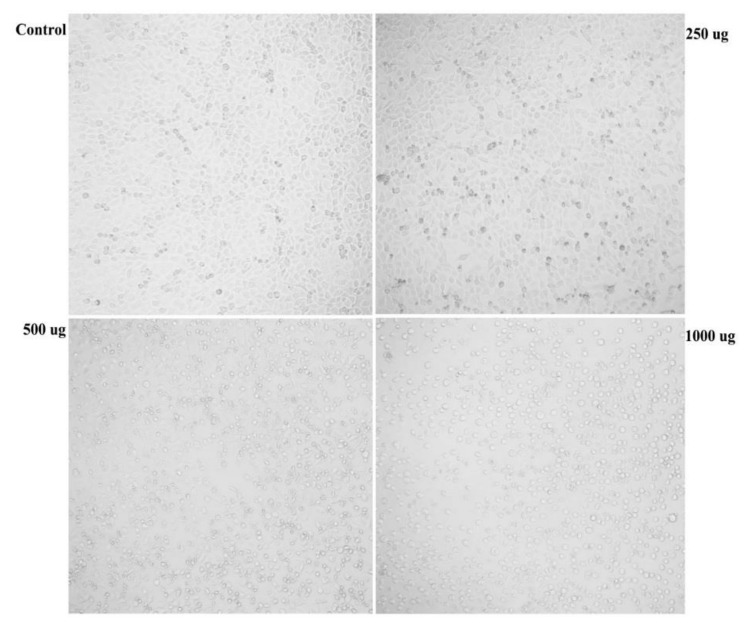
Morphological changes in A549 lung adenocarcinoma in response to different LLE doses.

Results of in vitro cytotoxic activities of LLE against A549 human lung adenocarcinoma (Figure 3) showed that the effect of the tested LLE serial dilutions was proportional to the applied concentration in both types of assays. The greatest decreases in cell viability (50.92 ± 0.5 and 75.78 ± 0.01%) were recorded when using 1000 μg/mL of LLE as detected by MTT and NRU assays, respectively. Furthermore, results showed that the IC_50_ of LLE against A549 cells was 297.31 and 841.23 µg/mL, obtained from NRU and MTT assays, respectively. The difference between both obtained IC_50_ values can be attributed to the fact that both assays act differently inside cancer cells. The MTT assay targets mainly the mitochondrial dehydrogenases, while the NRU assay measures lysosomal uptake of the dye. Our results also showed that the LLE exhibited no cytotoxicity against the normal non-tumorigenic BEAS-2B cells within the tested concentration range. Figure 4 indicates the morphological change occurring in A549 cells upon exposure to varying concentrations of LLE. It can be seen that cells treated with ≥ 250 μg/mL of LLE exhibit some morphological changes, which were concentration-dependent. 

Cells were diminished in size and became loosely attached to the plate surface. At higher LLE doses, cells were rounded up, flattened and they finally died. However, the morphological properties of control sets were nearly unchanged.

### 3.4. Body and Lung Weights

**Figure 5:**
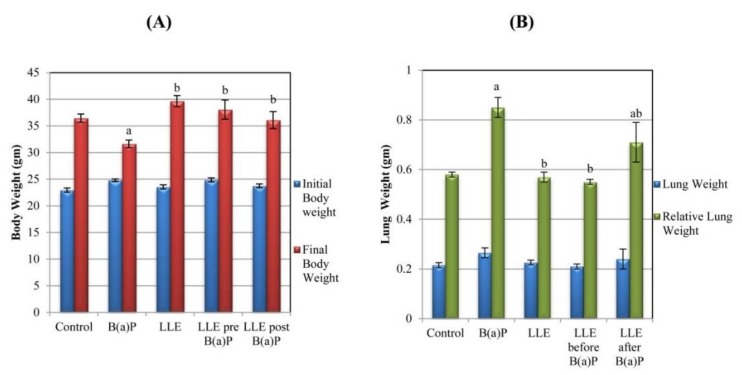
(**A**) Effect of LLE on the body weight, (**B**) Effect of LLE on the lung weight of different groups of mice. Each value is expressed as mean ± standard error (SE) for six mice in each group. ^a^ *p* < 0.05 statistically different from control group; ^b^ *p* < 0.05 statistically different from B(a)P group. Relative lung weight (lung weight/ final body weight) × 100.

Results (Figure 5A–B) showed that there was a significant decrease in the final body weight and significant increase in the relative lung weight of the B(a)P group compared to control group. Administration of LLE significantly increased the final body weight and significantly decreased lung weight in pre-treated and post-treated animals compared to the B(a)P group. There was not any marked change in body weight in animals administered LLE alone when compared to control animals.

### 3.5. Impact of L. Speciosa on Anti-Oxidation and Oxidation Markers

Our results (Table 3) revealed that a significant decrease in TAC, GSH level and CAT and GSH-Px activities with a concomitant increase in tissue MDA were observed in the B(a)P group as compared to control group. Treatment with LLE in pre-treated animals restored the changes in levels of TAC, GSH, CAT, GSH-Px and MDA to near-normal. LLE post-treated animals showed slightly increased TAC, GSH, CAT and GSH-Px activities, accompanied by decreased MDA level as compared to the B(a)P group. Animals in the LLE group showed no significant changes in antioxidant status when compared with the control group.

### 3.6. Impact of L. Speciosa on Tumor Marker Enzymes

The results (Table 4) revealed that AHH, ADA and LDH activities were significantly increased in the B(a)P group as compared to the control group. These activities were reversed to near-normal in the LLE pre-treated group. ADA and LDH activity levels in the LLE post-treated group were significantly decreased as compared to the B(a)P group, while AHH was slightly increased from the B(a)P group. No significant difference was observed between the LLE group and the control group.

### 3.7. Impact of L. Speciosa on MMP-2 and MMP-12 Activities

**Figure 6:**
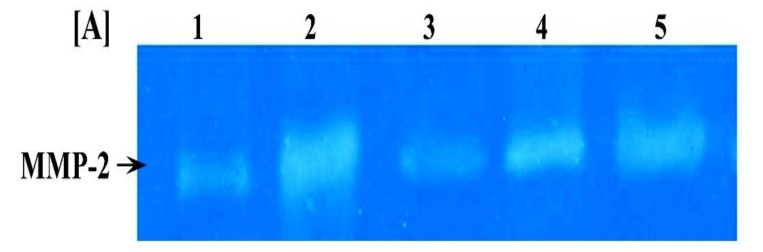

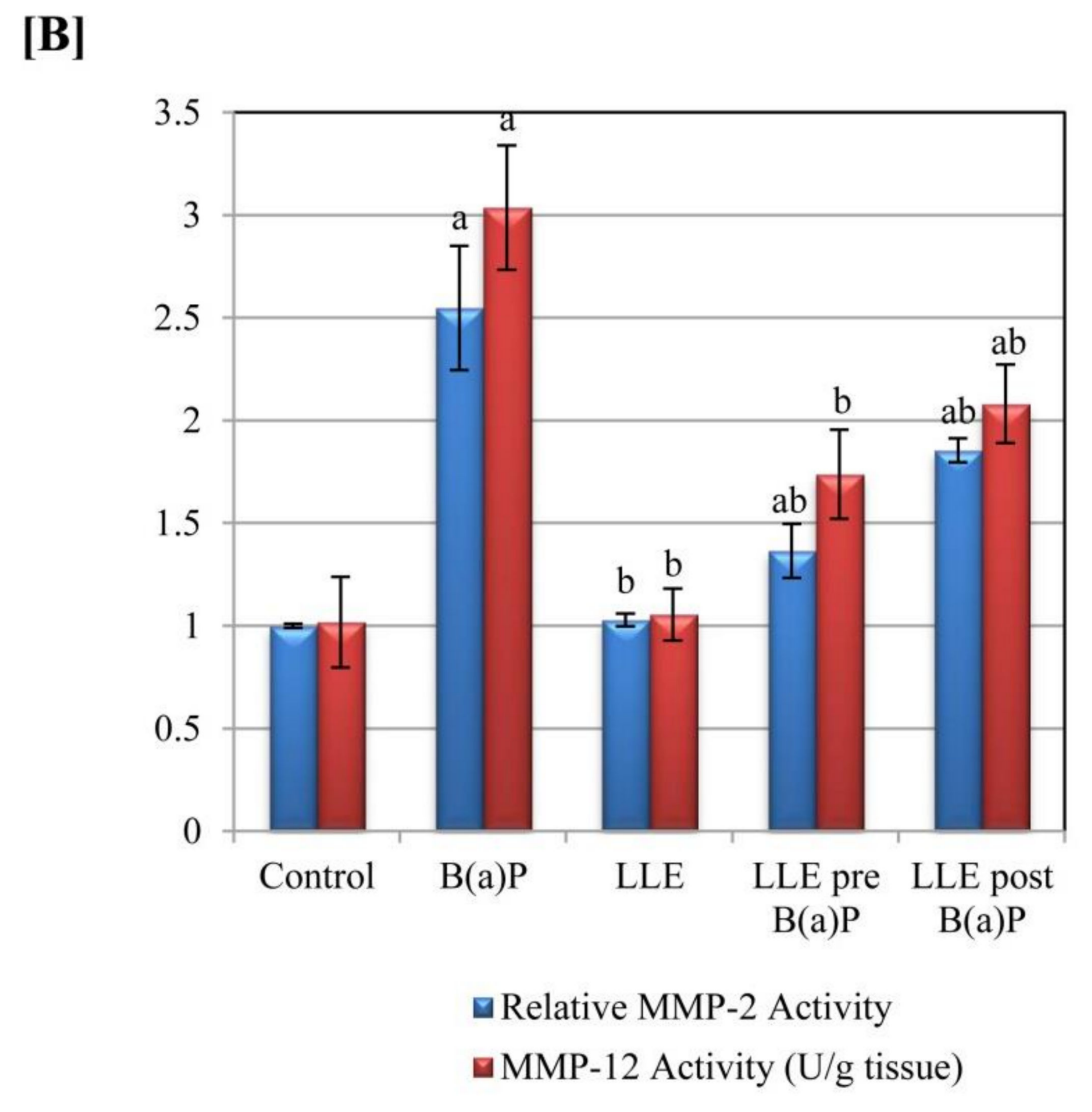
Detection of MMP-2 and MMP-12 activities in lungs of control and experimental mice. (**A**) SDS-PAGE gelatin zymogram of lung specimens from control group (lane 1), B(a)p group (lane 2), LLE group (lane 3), LLE pre-treated group (lane 4), and LLE post-treated group (lane 5). (**B**) Densitometric analysis of MMP-2 bands detected in gelatin zymograms, and MMP-12 activity. The bars represent mean ± SE for six mice in each group. ^a^*p* < 0.05 statistically different from control group; ^b^*p* < 0.05 statistically different from B(a)P group; ^ab^*p* < 0.05 vs both control and B(a)P groups.

To verify the effect of LLE on extracellular matrix (ECM) degradation in mice treated with B(a)P, MMP-2 and MMP-12 activities were estimated in THE lung tissue of control and experimental groups. Gelatin zymographic analyses of lung tissue samples, shown in Figure 5, reveal only one band corresponding to MMP-2. Gelatinolytic activity of MMP-2 significantly increased in the B(a)P group of animals compared with the control group. By contrast, LLE pre- and post-treated groups showed significant reduction compared to the B(a)P group. Moreover, the levels of MMP-2 in LLE and control groups were similar. A significant increase in MMP-12 activity was observed in the B(a)P group compared to the control group (Figure 6). This activity was restored to near-normal by the administration of LLE in pre- and post-treated groups. In addition, a significantly greater efficacy was observed in the pre-B(a)P group.

### 3.8. Impact of L. Speciosa on VEGF and NF-κB Levels

Our results (Table 5) revealed that administration of B(a)P led to a significant increase in the VEFG level as compared to the control group. Pre-treatment with LLE showed a complete reversal of the increased VEGF level. However, LLE treatment in the post-treated group resulted in a slight decrease compared to the B(a)P group. Concerning NF-κB levels, our results showed that the level of NF-κB was significantly elevated in the B(a)P group as compared to normal control. Pre-treatment with LLE significantly reversed the elevation in the NF-κB level; however, there is a slight difference in this NF-κB level between the post-treated and B(a)P groups (Table 5).

### 3.9. Expression of COX-2, TNF-α, and IL-6 Genes

**Figure 7:**
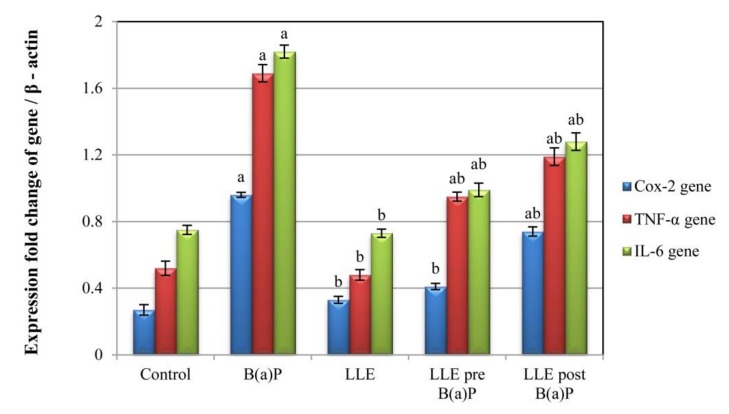
Expression of COX-2, TNF-α and IL-6 genes in the lung tissues of control and experimental mice relative to that of β-actin. Each value is expressed as mean ± SE for six mice in each group. ^a^*p* < 0.05 statistically different from control group; ^b^*p* < 0.05 statistically different from B(a)P group; ^ab^*p* < 0.05 vs both control and B(a)P groups.

From the results (Figure 7), expression levels of COX-2, TNF-α and IL-6 in the B(a)P group were significantly higher than those in the control group. On the other hand, expression levels of COX-2, TNF-α and IL-6 in the LLE-treated groups were significantly lower than those in the B(a)P group. Expression levels were lowest in the pre-treated group.

### 3.10. Expression of p53, BAX, Caspase-3, and Bcl-2 Genes

**Figure 8:**
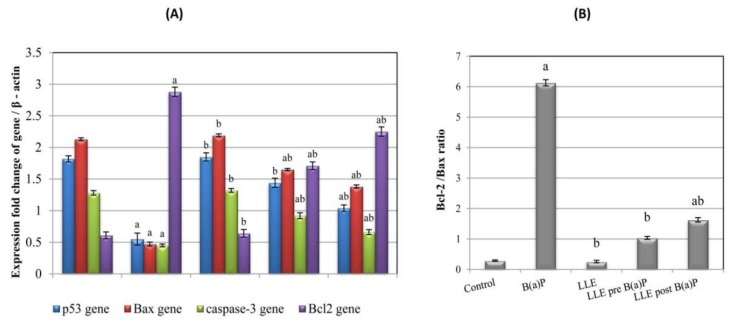
Effect of LLE on apoptotic process. (**A**): Expression of p53, Bax, caspase 3 and bcl-2 genes in the lung tissues of control and experimental mice relative to that of β- actin. (**B**): Bcl-2 /BAX ratio. Each value is expressed as mean ± SE for six mice in each group. ^a^*p* < 0.05 statistically different from control group; ^b^*p* < 0.05 statistically different from B(a)P group; ^ab^*p* < 0.05 vs both control and B(a)P groups.

Results revealed a significant decrease in the expression of p53, BAX and caspase-3, and a significant increase in the expression of Bcl-2 in the B(a)P group as compared to normal control. However, pre- and post-treatment with LLE resulted in a significant improvement in the expression of these genes as compared to the B(a)P group (Figure 8A). The Bcl-2/BAX ratio was also assessed. Our results revealed a significant increase in the Bcl-2/BAX ratio in the B(a)P group as compared to normal control. However, the Bcl2-/BAX ratio was significantly reduced by LLE pre- and post-treatments as compared to the B(a)P group (Figure 8B).

### 3.11. DNA Fragmentation

**Figure 9:**
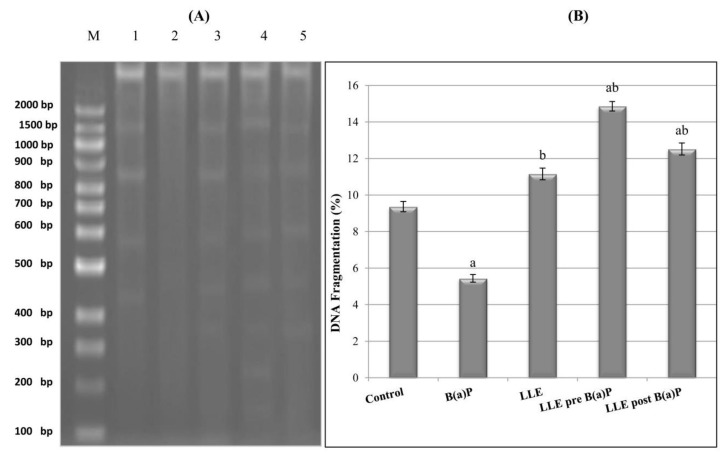
Effect of LLE on DNA fragmentation in lung tissues. (**A**): Densitometric analysis for DNA fragmentation using agarose gel electrophoresis. M: DNA ladder, Lane 1: Control group, Lane 2: B(a)P treated group, Lane 3: LLE group, Lane 4: LLE pre-treated group; and Lane 5: LLE post-treated group. (**B**): DNA fragmentation percentage. Each value is expressed as mean ± SE for six mice in each group. ^a^*p* < 0.05 statistically different from control group; ^b^*p* < 0.05 statistically different from B(a)P group; ^ab^*p* < 0.05 vs both control and B(a)P groups.

DNA fragmentation in the mouse genome was assessed in lung tissue (Figure 9A,B). The results revealed that DNA fragmentation decreased significantly following B(a)P treatment as compared to the control group. In contrast, DNA fragmentation increased significantly in the groups of mice pre-treated or post-treated with *L. speciosa* extract compared to the B(a)P group.

### 3.12. Pathological Examination

**Figure 10:**
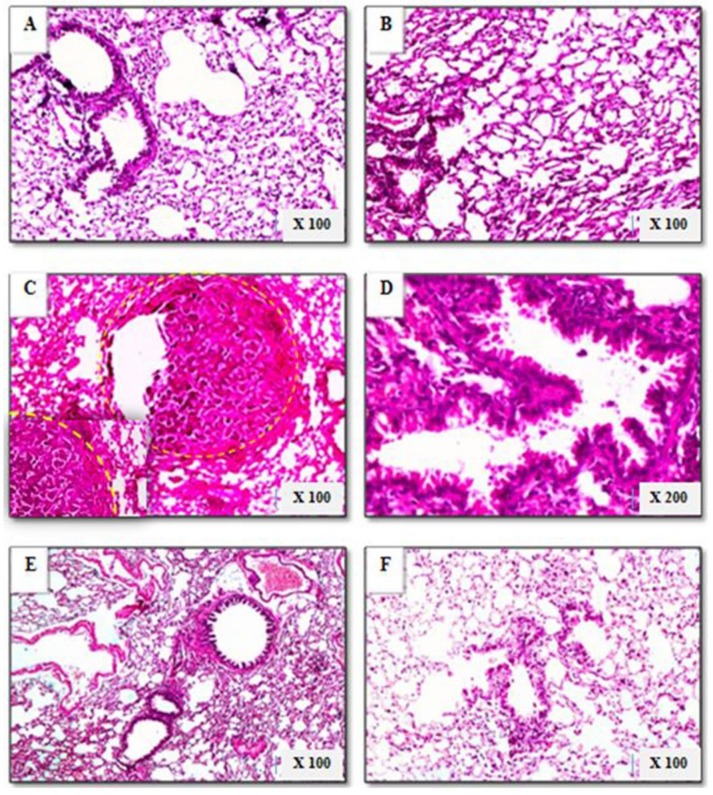
Histopathologic findings of lung sections in control and experimental animals (hematoxylin and eosin (H&E) sections). (**A**) Control mice showing normal uniform alveolar architecture. (**B**) *L. speciose*-treated mice showing normal alveolar pattern. (**C**, **D**) Mice treated with B(a)P alone; (**C**) shows well capsulated tumor formation (yellow circle) formed of irregular papillae lined by uniform epithelial cells, (**D**) shows dilated bronchioles with extensive papillary epithelial infoldings. (**E**) *L. speciosa* pre-treated mice showing greatly reduced alveolar damage. (**F**) *L. speciosa* post-treated mice showing small, large, and emphysematous alveoli, with some alveoli ruptured.

Histopathological findings encountered in control and experimental groups are shown in Figure 10. The control group displayed a normal histologic pattern in the form of generally uniform patent lung alveoli with intervening equally dispersed bronchioles. The *L. spesiosa* group showed a normal histologic pattern similar to the control group. In contrast, some sections from the B(a)P-treated group showed extensive alveolar changes in the form of well-defined capsulated tumors formed of irregular papillae lined by uniform cells with basal nuclei. Other sections showed diffuse dilated bronchioles with extensively irregular papillary infoldings. In the *L. spesiosa* pre-treated group there was decreased histopathologic damage compared to the B(a)P-treated group, in the form of small and large alveoli with intervening bronchioles exhibiting well-oriented epithelial cell lining. Additionally, in the *L. spesiosa* group, there was associated interstitial mild diffuse exudate of plasma cells and lymphocytes. 

The *L. spesiosa* post-treated group exhibited less extensive alveolar damage than the B(a)P-treated group, where alveoli were variable in shape and size (small, large, and emphysematous), and in some sections, ruptured. Intervening bronchioles showed extensive hyperplastic epithelial lining with some papillary infoldings. There were occasional peribronchial dense exudates of mature lymphocytes within the interstitium.

## 4. Discussion

Excessive production of reactive oxygen species (ROS) is implicated in the initiation, promotion and progression of cancer via induction of DNA mutations and modulation of various molecular pathways. Mitigation of oxidative stress is one promising strategy for chemoprevention to reduce the burden of cancer [41]. Antioxidants can overcome oxidative stress through their ability to scavenge free radicals, inhibit free-radical formation, and revise the antioxidant defense system [42]. However, due to adverse side effects associated with synthetic antioxidants, there is increasing interest in using natural antioxidants from plants instead of synthetic materials because of the ability of phytochemicals to reduce the risk of cancer without harmful side effects [43].

The *L. speciosa* plant has attracted great attention at the scientific level for its numerous pharmacological activities, particularly as an antioxidant [44]. However, no study has, to date, explored its anti-cancer effects. Therefore, the present study aimed to investigate antitumorigenic effects of this *L. speciosea* leaf extract.

The cytotoxic effects of LLE against A549 lung cancer cells caused significant decrease in cell viability in a concentration-dependent manner. Leaves of the *L. speciosa* plant are rich in polyphenolic compounds [12,45]. Anantharaju et al. [46] reported the cytotoxic effects of phenolic compounds against various cell lines. Polyphenols act as pro-oxidants by increasing intracellular production of reactive oxygen species, leading to the induction of apoptosis [47]. Hence, the cytotoxic effect of LLE on A549 cells may be a result of its polyphenolic content.

It is essential to confirm in vitro results with in vivo assays. In this study, B(a)P was used to induce lung tumorigenesis in a mouse model to investigate the ability of LLE to inhibit tumor development and progression. Kamaraj et al. [48] suggested that antioxidant status is a valuable tool for assessing the risk of oxidative damage-induced carcinogenesis, since antioxidants are the main line of defense against free radicals. Enzymatic antioxidants, such as CAT and GSH-Px, are responsible for scavenging and transforming free hydroperoxides and hydrogen peroxides into harmless molecules. GSH, one of the major non-enzymatic antioxidants, can directly neutralize hydroxyl radicals and maintain peroxides in their reduced form [49]. Liu et al. [50] reported that B(a)P can seriously perturb the antioxidant defense system and cause an excessive rise in lipid peroxidation during lung carcinogenesis. These deleterious effects are caused by enhanced oxidative stress and the generation of highly reactive free radicals [41,49]. Pre- and post-treatment with *L. speciosa* caused a reduction in lipid peroxidation along with an improvement in the antioxidant status in this study. This clearly indicates that LLE treatment reduces the oxidative stress induced by B(a)P, indicating one possible cytoprotective mechanism underlying the action of LLE against B(a)P-induced lung tumorigenesis. This was confirmed by the in vitro results of DPPH assays, which revealed the strong antioxidant potential of aqueous ethanolic LLE. The high content of total phenols and flavonoids in this extract could contribute to this antioxidant activity of LLE.

Administration of B(a)P caused significant elevation in the activities of tumor markers such as AHH, ADA and LDH. These tumor marker enzymes were previously reported to be elevated in lung cancer patients [51,52,53]. According to Moorthy et al. [54], elevated activity of AHH is essential in B(a)P metabolism, as it is the main enzyme responsible for B(a)P conversion to the extremely active benzo[a]pyren-7,8-dihydrodiol-9,10-epoxide, which is associated with tumor development and progression. Meanwhile, increased ADA activity may be a compensatory mechanism against the massive accumulation of its substrates (adenosine or deoxyadenosine), since increased proliferation leads to the salvage of nucleosides [55,56]. In addition, one possible reason for elevated levels of LDH could be its critical role in glycolysis [57]. Feng et al. [58] reported that accelerated glycolysis is an important feature of cancer cell metabolism, which supplies enough energy and biomaterials for proliferation.

Both pre- and post-treatment with LLE elicited a reduction in the activities of tumor marker enzymes. Inhibition of AHH activity could be attributed to the effect of LLE on the initiation phase of tumorigenesis. Reduction in ADA activity could reflect the ability of LLE to reduce purine turnover, and hence the tumor proliferation rate. 

Decreased LDH activity could demonstrate depletion of the energy needed to accelerate cancer cell growth due to decreased glycolysis. These results highlight the antiproliferative and antitumor properties of LLE.

Jabłońska-Trypuć et al. [59] and Mousa and Davis [60] reported that ECM degradation is critical for tumor growth and involved in all stages of cancer progression including invasiveness, angiogenesis and metastasis. MMPs are capable of degrading all ECM proteins, and their role as diagnostic and prognostic markers in many cancer types has been reported [61]. In fact, elevated levels of various MMPs have been detected in lung cancer. In the present study, the B(a)P-treated group showed an elevation in MMP-12 and MMP-2 activity. The elevated MMP-12 activity is in line with the findings of Houghton et al. [62]. According to Merchant et al. [63], MMP-12 as an oncogenic molecule that promotes lung cancer development through its major role in the inflammation process. Previously, Zaidi et al. [64] reported that elevated MMP-12 activity results in ECM degradation and release of growth factors to induce other MMPs at the gene and protein levels.

The elevated MMP-2 activity in the B(a)P-treated group is consistent with previous experimental findings of Anandakumar et al. [65]. According to Guo et al. [66] and Weng et al. [67], MMP-2 is a sensitive predictor of early-stage tumor invasion and metastasis of lung cancer. Further, Dong et al. [68] reported that MMP-2 promotes tumor cell proliferation and invasion through the positive regulation of VEGF expression.

Li et al. [69] stated that VEGF as a pro-angiogenic mediator activates vascular endothelial cells to stimulate extra MMP production. MMPs in turn digest the basement membrane of surrounding vessels, followed by cell proliferation and migration and formation of the endothelial capillary-like structure essential for tumor growth. This was in accordance with the results of Chakraborty et al. [70], who reported that VEGF is highly expressed in the tumor microenvironment and greatly promotes tumor angiogenesis. Data from the present study showed that B(a)P caused marked elevation in VEGF levels. These results are in agreement with the previous findings of Sultana [71], who reported that B(a)P enhances angiogenesis by elevating VEGF production.

B(a)P-mediated elevation in the levels of these markers was attenuated upon LLE treatment. Chan et al. [12] confirmed that ellagic acid is one of the major bioactive compounds isolated from the ethanolic leaf extract of *L. speciosa*. The anti-cancer activity of ellagic acid was previously reported in different kinds of cancers in both in vivo and in vitro studies [72]. According to Pitchakarn et al. [73], ellagic acid significantly reduced the proteolytic activity of MMP-2. Moreover, Wang et al. [74] demonstrated the ability of ellagic acid to suppress the VEGF receptor 2 signaling pathway through inhibiting its phosphorylation. Taken together, these findings indicate that LLE inhibits tumor progression and angiogenesis through reduction of MMP-2 activity and VEGF level.

Recently, Shi et al. [75] postulated that lung inflammation and B(a)P are partners in lung tumorigenesis. Cancer-related inflammation facilitates resistance to cell growth inhibition, escape from apoptosis, enhanced angiogenesis, tumor extravasation and finally metastasis [76]. According to David and Gooderham [77], B(a)P promotes inflammation via activating the transcriptional NF-κB pathway. In turn, activation of NF-κB leads to transcriptional upregulation of pro-inflammatory cytokines (IL-6 and TNF-α) and COX-2, consistent with our results. Therefore, attenuation of the levels of inflammatory markers is one of the targets during the chemoprevention of tumorigenesis. In the present study, the increased level of NF-κB and up-regulation of COX-2, TNF-α and IL-6 in the B(a)P-treated group was effectively attenuated by pre- and post-treatment with LLE. The targeting of different inflammatory markers by LLE may be attributed to its active polyphenolic compound, ellagic acid [78]. Umesalma et al. [79] reported that ellagic acid exerted its anti-inflammatory effect by inhibiting NF-κB activation, with subsequent down-regulation of COX-2, TNF-α and IL-6. Later, El-Shitany et al. [80] found that ellagic acid causes down-regulation of COX-2 expression through formation of hydrogen bonds with its active site. Thus, ellagic acid may inhibit inflammation by blocking the COX-2 receptor.

Numerous studies have implicated imbalance between cell proliferation and apoptosis in many pathological conditions. In fact, apoptosis has a central role in restricting tumor cell expansion, and apoptosis inhibition is pivotal to carcinogenesis [81].

Therefore, apoptosis-inducing activity could be a primary focus in verifying the effectiveness of a chemopreventive agent. In the present study, pre- and post-treatment with LLE-induced apoptosis, as evidenced by increased expression of p53 and the pro-apoptotic proteins Bax and caspase 3, and decreased expression of the anti-apoptotic protein Bcl-2 with a consequent drop in the Bcl-2/Bax ratio, one of the critical phenomena that regulate apoptosis. These results were further supported by the observed DNA fragmentation, which is a typical hallmark of apoptosis.

In the present study, the apoptotic activity of LLE in pre- and post-treated groups may be attributed to the involvement of ellagic acid in the mitochondrial apoptotic pathway [82], as evidenced by the increased expression of p53 and the pro-apoptotic proteins (Bax and caspase 3), and decreased expression of anti-apoptotic protein (Bcl-2) with a consequent drop in the Bcl-2/Bax ratio. Ellagic acid can also induce caspase-3-mediated apoptosis by increasing the Bax/Bcl-2 ratio [83]. Quercetin and ellagic acid synergistically induce apoptosis through p53 and the mitogen-activated protein kinases [84]. These results are further supported by DNA fragmentation.

Finally, it was observed that treatment with B(a)P resulted in reduced body weight; this may be due to nutritional privation, which causes adipose tissue and skeletal muscle wasting. In addition, the increase in relative lung weight observed in the B(a)P-treated group could be due to increased lung inflammation [30,41]. However, body weight loss and significant enlargement of the lung were completely prevented by pre- and post-treatment with LLE, due perhaps to its protective effect.

## 5. Conclusions

The findings of the present study highlight the efficacy of LLE in inhibiting B(a)P-induced lung tumorigenesis in Swiss albino mice. This tumor inhibition could be achieved through several pathways, including reduction in oxidative stress and modulation of inflammatory and apoptotic markers. Further evaluation of LLE is needed so that it can be explored as a novel chemopreventive agent in future preclinical trials for smokers at high risk of developing lung tumors.

## Figures and Tables

**Table 1:** Primer sequences used for qRT-PCR.

Gene	Primer Sequence (5′–3′)	Ref.
TNF-a	Forward: ACT GAA CTT CGG GGT GAT TG Reverse: GCT TGG TGG TTT GCT ACG AC	[36]
IL-6	Forward: GTC TAT ACC ACT TCA CAA GTC GGA Reverse: TTG GAT GGT CTT GGT CCT TAG CCA	[37]
P53	Forward: CGC AAA AGA AGA AGC CAC TA Reverse: TCC ACT CTG GGC ATC CTT	[38]
COX-2	Forward: CTG TAT CCC GCC CTG CTG GTG Reverse: ACT TGC GTT GAT GGT GGC TGT CTT	[39]
BAX	Forward: ACA AAG ATG GTC ACG GTC TGC C Reverse: GGT TCA TCC AGG ATC GAG ACG G	
Caspase-3	Forward: TGA GCA TTG ACA CAA TAC AC Reverse: AAG CCG AAA CTC TTC ATC	
Bcl-2	Forward: CTC AGT CAT CCA CAG GGC GA Reverse: AGA GGG GCT ACG AGT GGG AT	
b-actin	Forward: CAC GTG GGC CGC TCT AGG CAC CAA Reverse: CTC TTT GAT GTC ACG CAC GAT TTC	

**Table 2:** Phytochemical screening of *L. speciosa* leaf extract (LLE).

Phytochemicals	Present/Absent
Carbohydrates/glycosides	Present
Tannins	Present
Saponins	Present
Alkaloids	Absent
Anthraquinones	Absent
Phenols	Present
Flavonoids	Present
Triterpenes/steroids	Present

**Table 3:** Effect of *L. speciosa* extract on TAC serum level and GSH, CAT, GSH-Px and MDA levels in lung tissues of control and experimental mice.

Group	TAC (mM/L)	GSH (mg/g Tissue)	CAT (U/mg Tissue)	GSH -Px (U/g Tissue)	MDA (nM/g Tissue)
Control	0.92 ± 0.12	220.90 ± 4.492	17.15 ± 0.59	54.09 ± 1.20	219.93 ±10.70
B(a)P	0.42 ± 0.09^a^	125.19 ± 8.65^a^	12.56 ± 0.9^a^	31.77 ± 1.56^a^	427.23 ± 25.15^a^
% change from ctrl	54.34%	43.32%	26.76%	41.32%	94.25%
LLE	0.99 ± 0.06^b^	240.04 ± 10.40^b^	17.61 ± 1.06^b^	53.17 ± 6.52^b^	244.36 ±12.65^b^
LLE pre B(a)P	0.80 ± 0.06^b^	203.31 ± 8.28^b^	15.52 ± 0.89^b^	48.30 ± 3.38^ab^	305.27 ±19.74^ab^
% change from B(a)P	90.47%	62.40%	23.56%	52.03%	28.54%
LLE post B(a)P	0.72 ± 0.03^b^	160.37 ± 6.39^ab^	14.58 ± 0.48^a^	42.14 ± 2.11^ab^	366.48 ±15.37^ab^
% change from B(a)P	71.42%	28.10	16.08%	32.64%	14.21%

Each value is expressed as mean ± SE for six mice in each group. ^a^*p* < 0.05 vs control group; ^b^*p* < 0.05 vs B(a)P group; ^ab^*p* < 0.05 vs both control and B(a)P groups.

**Table 4:** Effect of *L. speciosa* extract on the activities of serum marker enzymes in control and experimental animals.

Group.	AHH (ng/ml)	ADA (pg/ml)	LDH (U/l)
Control	3.00 ± 0.10	121.33 ± 2.71	2304.40 ± 73.05
B(a)P	4.46 ± 0.36^a^	217.00 ± 19.42^a^	3756.10 ± 246.97^a^
% change from ctrl	48.66%	78.85%	62.99%
LLE	3.26 ± 0.26^b^	125.67 ± 5.30^b^	2133.00 ± 58.32^b^
LLE pre B(a)P	3.37 ± 0.13^b^	133.00 ± 11.08^b^	2406.90 ± 131.04^b^
% change from B(a)P	24.43%	38.70%	35.92%
LLE post B(a)P	3.74 ± 0.19^a^	151.00 ± 13.48^b^	2823.80 ± 102.89^ab^
% change from B(a)P	16.14%	30.41%	24.82%

Each value is expressed as mean ± S.E. for six mice in each group. ^a^p < 0.05 vs control group; ^b^p < 0.05 vs B(a)P group; ^ab^*p* < 0.05 vs both control and B(a)P groups.

**Table 5:** Effect of *L. speciosa* extract on the vascular endothelial growth factor (VEGF) in lung tissue and serum NF-κB levels in control and experimental mice.

Group	VEGF (pg/g Tissue)	NF-κB (ng/L)
Control	3102.13 ± 57.45	417.50 ± 42.40
B(a)P	3933.33 ± 208.01a	819.17 ± 89.16a
% change from ctrl	26.76%	96.20%
LLE	3026.70 ± 149.90b	486.66 ± 25.67b
LLE pre B(a)P	3226.70 ± 250.15b	508.33 ± 56.14b
% change from B(a)P	17.96%	37.94%
LLE post B(a)P	3453.30 ± 235.89	690.00 ± 78.18a
% change from B(a)P	12.20%	15.76%

Each value is expressed as mean ± SE for six mice in each group. ^a^*p* < 0.05 vs control group; ^b^*p* < 0.05 vs B(a)P group.
